# Nontoxigenic *Corynebacterium diphtheriae* Infections, Europe

**DOI:** 10.3201/eid2507.180995

**Published:** 2019-07

**Authors:** Aleksandra A. Zasada, Magdalena Rzeczkowska

**Affiliations:** National Institute of Public Health–National Institute of Hygiene, Warsaw, Poland

**Keywords:** nontoxigenic, *Corynebacterium diphtheria*, infection, ST8, bacteria, Europe, sequence type

**To the Editor:** We read with interest the article by Dangel et al. analyzing nontoxigenic *Corynebacterium diphtheriae* infections in northern Germany during 2016–2017 (*1*). Among the cases, 2 patients originated from Poland; each experienced an invasive disease, 1 endocarditis and 1 sepsis. Poland and Germany are neighboring countries. In Poland, we also observed an accumulation of nontoxigenic *C. diphtheriae* infections during 2016–2017. In both countries, most infections were caused by isolates belonging to sequence type (ST) 8 biotype gravis, which we previously suspected of having increased pathogenic properties (*2*). 

ST8 has been causing infection in Poland since 2004 and was isolated in Russia before that (*2*,*3*). However, the first ST8 isolate was not obtained in northern Germany until 2015, suggesting spread of pathogenic ST8 from eastern to western Europe. Comparing epidemiologic data from Poland during 2012–2017, we confirmed 48 cases of nontoxigenic *C. diphtheriae*, increasing from 3 cases in 2012 to 20 in 2017. As seen in northern Germany, most affected patients in Poland were male (>80%), and ≈30% of patients were homeless, alcohol addicted, or both. We did not identify HIV as a risk factor. We saw a sharp increase in cases during the time of the Dangel et al. report as well, from 10 cases in 2016 to 20 in 2017. Nevertheless, in Poland, 40% of isolates (19/48) during 2012–2017 were obtained from invasive infections, whereas in Germany only 9 isolates (≈12%) were obtained from cases with severe invasive complications. None of the cases in Poland were related epidemiologically.

We hypothesize that pathogenic ST8 could spread to other countries in Europe in a few years and that persistence of ST8 isolates in the population might be related to increases in the number of invasive infections. The scale of the problem of nontoxigenic *C. diphtheriae* infections in Europe remains unknown because only toxigenic infections are registered. Lack of registration leads to lack of prevention and, thus, to outbreak development and spread.
